# *Borrelia burgdorferi bb0426* encodes a 2′-deoxyribosyltransferase that plays a central role in purine salvage

**DOI:** 10.1111/j.1365-2958.2009.06740.x

**Published:** 2009-05-26

**Authors:** Kevin A Lawrence, Mollie W Jewett, Patricia A Rosa, Frank C Gherardini

**Affiliations:** Laboratory of Zoonotic Pathogens, Rocky Mountain Laboratories, National Institute of Allergy and Infectious Diseases, National Institutes of HealthHamilton, MT 59840, USA

## Abstract

*Borrelia burgdorferi* is an obligate parasite with a limited genome that severely narrows its metabolic and biosynthetic capabilities. Thus survival of this spirochaete in an arthropod vector and mammalian host requires that it can scavenge amino acids, fatty acids and nucleosides from a blood meal or various host tissues. Additionally, the utilization of ribonucleotides for DNA synthesis is further complicated by the lack of a ribonucleotide reductase for the conversion of nucleoside-5′-diphosphates to deoxynucleosides-5′-diphosphates. The data presented here demonstrate that *B. burgdorferi* must rely on host-derived sources of purine bases, deoxypurines and deoxypyrimidines for the synthesis of DNA. However, if deoxyguanosine (dGuo) is limited in host tissue, the enzymatic activities of a 2′-deoxyribosyltransferase (DRTase, encoded by *bb0426*), IMP dehydrogenase (GuaB) and GMP synthase (GuaA) catalyse the multistep conversion of hypoxanthine (Hyp) to dGMP for DNA synthesis. This pathway provides additional biochemical flexibility for *B. burgdorferi* when it colonizes and infects different host tissues.

## Introduction

The synthesis of nucleic acids is critical in all living organisms as a means of storing and processing information for all cellular functions. Likewise, the production and/or assimilation of purine and pyrimidine bases for nucleic acid synthesis are also essential. Organisms with complete purine and pyrimidine biosynthetic pathways are capable of producing nucleotides *de novo* and do not require an external source of nucleic acid intermediates. Conversely, some pathogenic bacteria lack a complete set of functional enzymes to catalyse nucleotide synthesis and must rely on salvage pathways that require host-derived sources of nucleotide precursors (Nygaard, 1993). Such obligate parasites must generally have, at minimum, a core set of essential enzymes to perform basic purine/pyrimidine salvage metabolism. A critical enzyme in either *de novo* or salvage pathways is the ribonucleotide reductase (RNR), which enzymatically reduces ribonucleotides to their deoxy-analogues and maintains an adequate deoxynucleotide pool, through allosteric regulation, for efficient incorporation into DNA. It is widely thought that these enzymes are central to even the most basic nucleotide pathway and critical for all living organisms, regardless of their capabilities for purine/pyrimidine biosynthesis (Nordlund and Reichard, 2006).

*Borrelia burgdorferi*, the etiological agent of Lyme disease, is an obligate parasite with a 1.5-mega-base genome (Fraser *et al*., 1997), and limited metabolic and biosynthetic capacity. Survival in its arthropod vector and mammalian host requires the organism to ‘scavenge’ amino acids, fatty acids and nucleosides for the biosynthesis of proteins, lipids/lipoproteins and nucleic acids respectively. The success of this bacterium is also dependent upon making efficient use of ribonucleotides for DNA synthesis in the absence of a functional RNR. This deficiency limits the biochemical options for DNA synthesis in *B. burgdorferi* to either acquiring all deoxynucleosides from the host, potentially limiting the available sites for host colonization and replication (e.g. *Ureaplasma urealyticum*) (Carnrot *et al*., 2003) and/or biochemically modifying nucleosides to provide the necessary deoxynucleosides that are not available from the host(s). In this report, we present data that suggest that *B. burgdorferi* both requires deoxypurines and deoxypyrimidines from its host and makes use of a 2′-deoxyribosyltransferase to interconvert purine bases to deoxynucleosides when host tissue sources of some deoxynucleosides are limiting.

## Results

### *B. burgdorferi* transports and incorporates nucleosides and deoxynucleosides

The genome of *B. burgdorferi* does not harbour the genes encoding enzymes for *de novo* purine and pyrimidine biosynthesis. Furthermore, *B. burgdorferi* is deficient in the genes encoding central components of the ‘classic’ purine salvage pathway, such as hypoxanthine-guanine phosphoribosyltransferase (Hpt), adenylosuccinate synthase (PurA) and adenylosuccinate lyase (PurB), which have been described in *Borrelia hermsii, Borrelia duttonii* and *Borrelia recurrentis* (Barbour *et al*., 2005; Pettersson *et al*., 2007; Lescot *et al*., 2008). In addition, it has been recently demonstrated that *B. burgdorferi* does not contain the gene operon for a RNR (Fraser *et al*., 1997; Barbour *et al*., 2005; Pettersson *et al*., 2007). These data suggest that *B. burgdorferi* must require exogenous sources of nucleosides and deoxynucleosides for RNA and DNA synthesis.

To investigate this, *B. burgdorferi* cells were assayed for their ability to transport and incorporate [^3^H]-labelled nucleoside-5′-monophosphates (NMP) or deoxynucleoside-5′-monophosphates (dNMP) into nucleic acids. The NMPs and dNMPs were chosen for these experiments because they are likely available in the tick midgut and saliva during blood meal digestion and in host tissue at the initial site of infection as a result of host cells lysis and subsequent nucleic acid degradation due to the innate immune response. However, NMPs and dNMPs are not generally transported across cell membranes (Nygaard, 1993). Instead, 5′nucleotidases in the cell membrane specifically dephosphorylate NMPs or dNMPs to nucleosides or deoxynucleosides to facilitate transport (Yagil and Beacham, 1975). Genome analysis indicates that *B. burgdorferi* harbours a putative 5′-nucleotidase (*bb0504*) that contains a catalytic HD domain (a family of metal-dependent phosphohydrolases) (Aravind and Koonin, 1998) and a transmembrane domain (ExPASy server) (Gasteiger *et al*., 2003). Furthermore, a putative nucleoside ABC transporter system, encoded by a three-gene operon (BB0677–79), has also been identified in *B. burgdorferi* (Overbeek *et al*., 2003). Thus, we believe that NMPs and dNMPs would be available during critical stages of the infective cycle and effectively dephosphorylated and transported for incorporation into RNA and DNA.

**Fig. 1:**
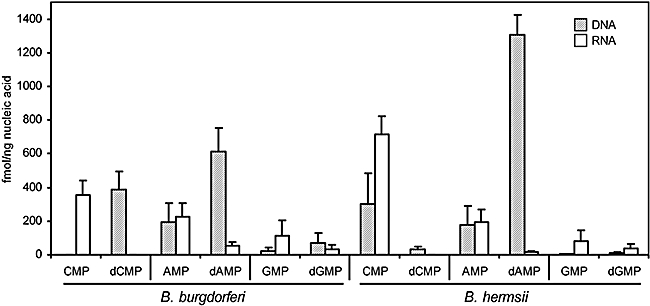
Nucleotide incorporation by *B. burgdorferi* and *B. hermsii* using a variety of [^3^H]-labelled ribonucleotide and deoxyribonucleotide pairs as substrates. Error bars represent the standard deviation of triplicate samples, normalized to either DNA or RNA yield. Femtomoles of [^3^H]-labelled nucleotide incorporated were calculated from the counts per min (cpm) and the specific activity of the labelled substrate.

In the initial experiments, *B. burgdorferi* cells were grown in Barbour-Stoenner-Kelly (BSK)II medium containing [^3^H]-cytosine monophosphate (CMP) or [^3^H]-deoxycytosine monophosphate (dCMP), followed by isolation of RNA and DNA and quantification of incorporated [^3^H] by scintillation counting. The results are shown in Fig. 1 (columns labelled CMP and dCMP). When *B. burgdorferi* cells were grown in the presence of [^3^H]-CMP, the radiolabel was detected in the RNA (Fig. 1, ∼400 fmol ng^−1^ of nucleic acid), but was not detected in purified DNA. Conversely, when cells were grown in the presence of [^3^H]-dCMP, the radiolabel was detected in isolated DNA (Fig. 1, ∼400 fmol ng^−1^ of nucleic acid), but not in purified RNA. These data indicated that *B. burgdorferi* was not able to convert cytosine to deoxycytosine for incorporation into DNA. In contrast, when *B. hermsii* cells were grown in the presence of [^3^H]-CMP, the radiolabel was detected in both RNA and DNA (Fig. 1, column labelled CMP; ∼700 and 300 fmol ng^−1^ of nucleic acid respectively), indicating that *B. hermsii* is able to convert cytosine to deoxycytosine for DNA synthesis. These results were not surprising as a RNR, for converting ribonucleotide diphosphate to 2′-deoxyribonucleotide diphosphate, has been identified in *B. hermsii* (Barbour *et al*., 2005; Pettersson *et al*., 2007). These data provided experimental evidence for a functionally active RNR in *B. hermsii* and the absence of such in *B. burgdorferi*.

To further assess the ability of *B. burgdorferi* to transport and incorporate nucleic acid precursors, *B. burgdorferi* cells were grown in the presence of [^3^H]-labelled purines (AMP, dAMP, GMP or dGMP) and the incorporation of these nucleosides was assayed as described above and the results are shown in Fig. 1 (columns labelled AMP, dAMP, GMP and dGMP respectively). Surprisingly, when cells were grown in the presence of [^3^H]-AMP, the radiolabel was detected in both DNA and RNA (∼200 fmol ng^−1^ of nucleic acid in RNA and DNA). Moreover, radiolabel was detected in DNA (∼600 fmol ng^−1^ of nucleic acid) and RNA (∼50 fmol ng^−1^ of nucleic acid) when cells were grown in the presence of [^3^H]-dAMP. Similarly, when the cells were grown in the presence of [^3^H]-GMP or [^3^H]-dGMP, the radiolabel was detected in both the DNA and RNA (∼50 and 100 fmol ng^−1^ of nucleic acid, respectively, for GMP and 100 and 50 fmol ng^−1^ of nucleic acid, respectively, for dGMP). In comparison, when *B. hermsii* cells were grown in the presence of [^3^H]-AMP, the radiolabel was detected in both the DNA and RNA (Fig. 1, ∼200 fmol ng^−1^ of nucleic acid for RNA and DNA) at levels similar to those observed in *B. burgdorferi.* As expected, [^3^H]-dAMP was incorporated almost exclusively into DNA (Fig. 1, ∼1400 fmol ng^−1^ of nucleic acid in DNA compared with ∼10 fmol ng^−1^ of nucleic acid in RNA). When *B. hermsii* cells were grown in the presence of [^3^H]-GMP or [^3^H]-dGMP, the levels of incorporation of [^3^H] were similar to those observed in *B. burgdorferi.* It is important to note that the levels of incorporation of [^3^H]-GMP and [^3^H]-dGMP in both bacteria were much lower than those observed with [^3^H]-AMP or [^3^H]-dAMP, suggesting that *B. burgdorferi* and *B. hermsii* were inefficient at transporting and/or incorporating guanosine (Guo) and deoxyguanosine (dGuo). These data suggested that in the absence of a RNR, an alternative route for generating reduced ribonucleotides for purine metabolism must be present in *B. burgdorferi* and an alternate biochemical pathway must exist to supplement inefficient Guo and dGuo transport in *B. burgdorferi,* distinct from that described in *B. hermsii* (Barbour *et al*., 2005; Pettersson *et al*., 2007).

### BB0426 encodes a purine-specific 2′-deoxyribosyltransferase

**Fig. 2:**
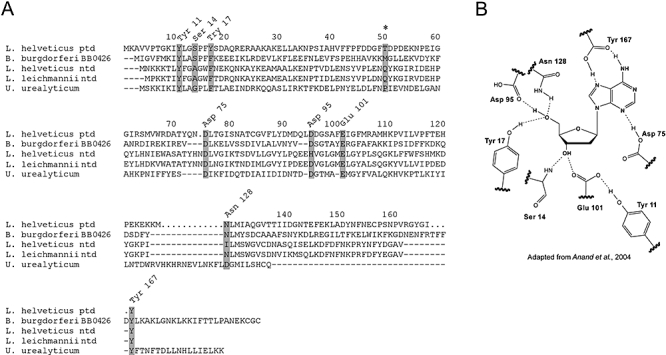
Amino acid alignment of the 2′-deoxyribosyltransferase of *Lactobacillus* species, *Ureaplasma* and *B. burgdorferi* BB0426. A. Alignment of *Lactobacillus helveticus* DRTase I active site residues (indicated by shaded boxes) and the catalytic residue unique to DRTase II (asterisk) (Anand *et al*., 2004) with the sequences of BB0426 and some other members of Pfam 05014 (Finn *et al*., 2006). B. Spatial arrangement of amino acid residues involved in deoxynucleoside binding with an dAdo molecule shown in bold (Anand *et al*., 2004).

**Fig. 3:**
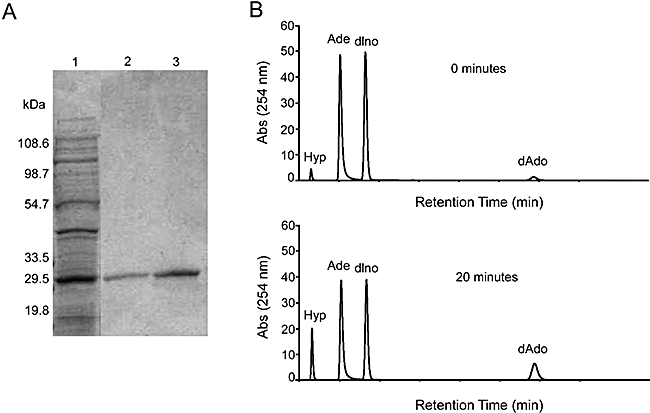
Purification and characterization of BB0426_opt_. A. Commassie-stained SDS-PAGE gel with (1) *E. coli* lysate expressing BB0426_opt_ and eluted fraction from a T7 affinity column. B. HPLC chromatograms showing the enzymatic activity of BB0426_opt_. In this assay, deoxyinosine (dIno) served as the deoxyribosyl donor and adenine (Ade) the deoxyribosyl acceptor: deoxyadenosine (dAdo) and hypoxanthine (Hyp) are the products of the reaction.

Close inspection of the *B. burgdorferi* genome revealed a plausible explanation for the RNR-like biochemical activity observed in the purine incorporation assay. While initial genome analyses had provided no evidence for this activity, a sequence search using Pfam (Finn *et al*., 2006) identified BB0426 as a potential member of a group of 2′-deoxyribosyltransferases (DRTases) (PF05014) that are involved in nucleoside/deoxynucleoside recycling in *Lactobacillus* species (Fig. 2) (Kaminski, 2002). The basis for this match was centred on near-identical active site residues that have been determined to be critical for substrate binding and enzyme catalysis (Fig. 2B) (Anand *et al*., 2004). There are two classes of DRTases: DRTase I enzymes catalyse the transfer of a 2′-deoxyribose moiety between purine bases exclusively, while DRTase II enzymes can also transfer a 2′-deoxyribose between two pyrimidines or between pyrimidines and purines. To determine the biochemical properties of BB0426, an optimized *bb0426* was synthesized using the codon preferences for expression in *Escherichia coli* (GeneArt). The optimized gene, *bb0426-opt*, was cloned into pET17b and the T7-tagged, recombinant protein was purified to apparent homogeneity (Fig. 3A).

To measure deoxyribosyltransferase activity, the purified protein was combined with various substrates to serve as either donors or acceptors of the deoxyribose moiety and the reactions monitored by HPLC at an absorbance of 254 nm (Fig. 3B). Control assays were performed with heat-inactivated enzyme. Initial assays which used deoxyinosine (dI) as the deoxyribose donor and adenine (Ade) as the deoxyribose acceptor resulted in the generation of deoxyadenosine (dAdo) and hypoxanthine (Hyp), with a specific activity determined to be 42 ± 4.9 nmol min^−1^ mg^−1^ (Table 1). Figure 3B is a representative HPLC chromatogram showing the conversion of Ade to dAdo when dI was used as a donor. At reaction time of 0 min (t_0_), the A_240_ of Ade and dI was approximately 50 milliabsorbance units (mAU). At reaction time of 20 min (t_20_), the absorbance of both had decreased to approximately 40 mAU. The decrease in Ade and dI concentrations corresponded to a subsequent increase in the absorbance of dAdo (Fig. 3B: from ∼1 mAU at t_0_ to ∼10 mAU at t_10_) and of Hyp (the product generated from the removal of the deoxyribosyl moiety from dI), from ∼5 mAU at t_0_ to ∼20 mAU at t_10_. Other purines were used as deoxyribose donors (i.e. dAdo and dGuo) and acceptors Hyp, guanine (Gua) and Ade, and the specific activity of BB0426_opt_ with each potential deoxyribose donor was measured by HPLC. The results are shown in Table 1. The specific activities range from 16 ± 2.5 nmol min^−1^ mg^−1^ (dAdo to Hyp) to 94 ± 2.4 nmol min^−1^ mg^−1^ (dAdo to Gua) and indicated that BB0426_opt_ was able to effectively transfer the deoxyribose from any deoxypurine to any purine base. However, purified BB0426_opt_ was not able to transfer the deoxyribosyl moiety from purine donors to pyrimidines (e.g. dI to cytosine) or between pyrimidines (e.g. deoxythymidine to cytosine) (Table 1). Taken together, these results indicate that BB0426 is a member of the purine-specific DRTase I subfamily and the activity of BB0426 could explain the observed incorporation of purine nucleosides into DNA.

**Table 1:** Specific activities of BB0426_opt_ with various substrates compared with DRTase I^a^ and DRTase II^a^ from *Lactobacillus helveticus* (Kaminski, 2002).

			Specific activity (nmol min^−1^ mg^−1^)		
			*B. burgdorferi*	*L. helveticus*	
Deoxyribose donor	Acceptor	Enzymatic product	BB0426	DRTase I	DRTase II
Purines					
Deoxyinosine	Adenine	Deoxyadenosine	42 ± 1.2^b^	83	3
Deoxyinosine	Guanine	Deoxyguanosine	45 ± 4.9	–	–
Deoxyadenosine	Hypoxanthine	Deoxyinosine	16 ± 2.5	61	8
Deoxyadenosine	Guanine	Deoxyguanosine	94 ± 2.4	–	–
Deoxyguanosine	Adenine	Deoxyadenosine	85 ± 10.2	50	16
Deoxyguanosine	Hypoxanthine	Deoxyinosine	18 ± 0.7	54	3
Deoxyadenosine monophosphate	Hypoxanthine	Deoxyinosine	< 1	–	–
Deoxyguanosine monophosphate	Hypoxanthine	Deoxyinosine	< 1	–	–
Pyrimidines					
Deoxythymidine	Cytosine	Deoxycytosine	< 1	< 1	28
Deoxycytosine	Thymine	Deoxythymidine	2	< 1	32
Purine/pyrimidine					
Deoxyinosine	Cytosine	Deoxycytosine	< 1	< 1	8
Deoxyadenosine	Cytosine	Deoxycytosine	< 1	< 1	34

a DRTase I or II: deoxyribosyltransferase class I or II.

b Mean values and standard deviations represent triplicate analyses.

### The fate of hypoxanthine in *B. burgdorferi*

**Fig. 4:**
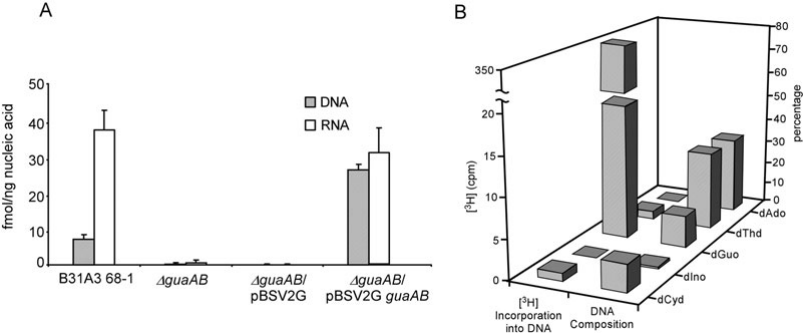
[^3^H]-Hyp conversion and incorporation into nucleic acids. A. [^3^H]-Hyp conversion and incorporation into the RNA and DNA of strains B31A3 68-1, Δ*guaAB*, Δ*guaAB*/pBSV2G (vector control) and Δ*guaAB*/pBSV2G *guaAB* (complement). Femtomoles of [^3^H]-Hyp incorporated were calculated from the counts per minute (cpm) and the specific activity of the labelled substrate. Error bars represent the standard deviations of triplicate samples. B. HPLC analyses of the incorporation of [^3^H] into individual deoxynucleosides of DNA isolated from [^3^H]-Hyp labelled *B. burgdorferi* cells. The left side of the *z*-axis, [^3^H]-Hyp was determined to be incorporated primarily as dGuo. The right of the *z*-axis shows the per cent composition of unlabelled DNA as a control to verify that DNA digestion went to completion.

Previously, it was demonstrated that *B. hermsii,* a closely related member of the *Borrelia* genus, has a classic purine salvage pathway, including genes encoding a class Ib RNR (Barbour *et al*., 2005; Pettersson *et al*., 2007). Furthermore, it was shown that *B. burgdorferi* is able to incorporate [^3^H]-Hyp into RNA and DNA (Pettersson *et al*., 2007). This result was puzzling as *B. burgdorferi* lacked a hypoxanthine phosphoribosyltransferase (Hpt), an adenylosuccinate synthase (PurA) and an adenylosuccinate lyase (PurB), which have a suggested involvement in the incorporation of Hyp into RNA and DNA in *B. hermsii* (Pettersson *et al*., 2007)*.* However, the activity of BB0426_opt_ (e.g. Hyp + dAdo yielded dI + Ade) might explain these data. To determine the fate of Hyp in *B. burgdorferi*, cells were grown in BSKII containing [^3^H]-Hyp, the RNA and DNA purified and assayed for radioactivity as described above. The results are shown in Fig. 4A (column labelled B31A3 68-1). The amount of [^3^H]-Hyp incorporated into the DNA was ∼8 fmol ng^−1^ of nucleic acid, while the amount incorporated into RNA was approximately 5× that level at ∼38 fmol ng^−1^ of nucleic acid. As previously observed, these data confirmed that Hyp was incorporated into the nucleic acids of *B. burgdorferi* even in the absence of an Hpt. One explanation for the incorporation of Hyp into RNA is due to the presence of a xanthine-guanine phosphoribosyltransferase (*gpt*, BB0103) (Table 2), which could potentially convert Hyp to IMP, and then be converted to GMP by IMP dehydrogenase (GuaB) (Zhou *et al*., 1997) and GMP synthase (GuaA) (Margolis *et al*., 1994) for incorporation into RNA. However, the presence of [^3^H]-label in the DNA of *B. burgdorferi* was difficult to explain. To determine the specific base containing the [^3^H]-label in the purified DNA, the DNA from the [^3^H]-Hyp incorporation assays was digested with nuclease, dephosphorylated, and the deoxynucleosides fractionated by HPLC. These results are shown in Fig. 4B. According to these data, > 99% of the [^3^H]-label was detected in dGuo, indicating that Hyp was being converted to dGMP for incorporation into the DNA of *B. burgdorferi.*

**Table 2:** Enzymes involved in *B. burgdorferi* purine salvage pathway.

Enzyme name	Gene designation	Open reading frame	Reference
Adenine deaminase	*adeC*	*bbk17*	Jewett *et al*. (2007b)
Adenine phosphoribosyltransferase	*apt*	*bb0777*	Overbeek *et al*. (2003)
Adenylate kinase	*adk*	*bb0417*	Overbeek *et al*. (2003)
Deoxynucleoside kinase	*dgk*	*bb0239*	Overbeek *et al*. (2003)
Deoxyribosyltransferase	*ptd*	*bb0426*	Finn *et al*. (2006)
GMP synthase	*guaA*	*bbb18*	Margolis *et al*. (1994)
IMP dehydrogenase	*guaB*	*bbb17*	Zhou *et al*. (1997)
Guanine-hypoxanthine permease	*pbuG*	*bbb22–23*	Byram *et al*. (2004)
Nucleoside transport system (ABC)	*mglA, mglC1, mglC2*	*bb0677–79*	Overbeek *et al*. (2003)
Nucleotidase	–	*bb0504*	Gasteiger *et al*. (2003)
Nucleoside phosphorylase	–	*bb0588, bb0375, bbi06*	Overbeek *et al*. (2003)
Nucleoside diphosphate kinase	*ndk*	*bb0463*	Overbeek *et al*. (2003)
Xanthine-guanine phosphoribosyltransferase	*gpt*	*bb0103*	Pettersson *et al*. (2007)

**Fig. 5:**
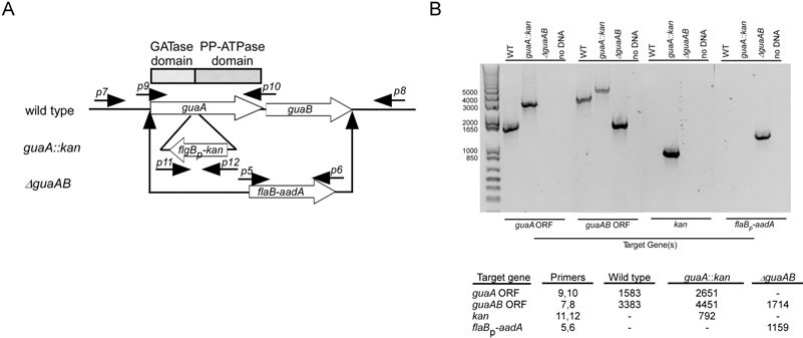
Characterization of *guaA*::*kan* and Δ*guaAB* mutant strains. A. Diagram of gene organization for wild-type *guaAB*, as well as the insertion points of antibiotic-resistance cassettes for the two mutant strain constructs (*guaA*::*kan* and Δ*guaAB*). The positions of the primers used for PCR are identified by number and correspond to those listed in Table 3. The location of GATase and PP-ATPase catalytic domains in *guaA* are indicated by rectangular boxes. B. Agarose gel containing PCR products using genomic DNA from the specified *B. burgdorferi* strains and primer sets corresponding to the target gene of interest. Standards in base pairs are shown on the left. Predicted product size (in base pairs) for each primer pair is indicated in the inset table.

Given these results, it appeared likely that [^3^H]-Hyp incorporation into *B. burgdorferi* DNA was due to the enzymatic activity of BB0426. Purified BB0426_opt_ was able to transfer the deoxyribosyl moiety from dAdo (or dGuo) to Hyp, yielding dI (Table 1). We then speculated that dI could be phosphorylated to dIMP and converted to dGTP by IMP dehydrogenase (GuaB), GMP synthase (GuaA), deoxyguanosine kinase (Dgk) and nucleoside diphosphate kinase (Ndk). To test this hypothesis, a GuaA mutant (*guaA*::*kan*), in which an antibiotic-resistance cassette was inserted 559 bp downstream of the *guaA* start codon resulting in a 229 bp deletion (Fig. 5A), was examined for the ability to incorporate [^3^H]-Hyp. Unlike wild-type *B. burgdorferi*, the digested purified DNA isolated from strain *guaA*::*kan* cells, grown in the presence of [^3^H]-Hyp, contained no [^3^H]-dGuo, indicating that this mutant was unable to biochemically convert [^3^H]-Hyp to [^3^H]-dGTP (data not shown). However, this mutant did incorporate [^3^H]-Hyp into DNA as [^3^H]-dAdo (data not shown), an activity not present in the wild-type bacteria.

**Table 3:** *B. burgdorferi* strains and primers used in this study.

Clone	Genotype	Plasmids lost	Reference
*B. hermsii* DAH	Wild-type	Unknown	Hinnebusch *et al*. (1998)
B31A3 68-1	Parent strain	cp9, lp25, lp56	This study
B31A3 m9	Parent strain	cp9, lp21	Tilly et al. (2004)
*guaA*::*kan*	A3 M9 *guaA*:: *flgB*_p_-*kan*	cp9, lp21	This study
Δ*guaAB*	A3 68-1 *guaAB*::*flaB*_p_-*aadA*	cp9, lp25, lp56	This study
Δ*guaAB*/pBSV2G	A3 68-1 *guaAB*::*flaB*_p_-*aadA*	cp9, lp25, lp56	This study
ΔguaAB/pBSV2G *guaAB*	A3 68-1 *guaAB*::*flaB*_p_-*aadA*/ pBSV2G *guaAB*	cp9, lp25, lp56	This study
Number	Primer name	Sequence	

p1	*guaB*−500	5′-TGCTGATCCTATGACGTTCTTAAGC-3′	
p2	*guaA*+500	5′-TAGGGTTGATATTGCATAAGCTCCCGC-3′	
p3	*guaB* 13852-SalI 3′	5′-GCGTCGACAAAAGATCAAAATATTGCATCCTTC-3′	
p4	*guaA* 16727-SalI 5′	5′-GCGTCGACAGCATATTTGGCTTTGCTTATGTCG-3′	
p5	*flaB-*XhoI 5′	5′-CCGCTCGAGCTGTCGCCTCTTGTGGCTTC-3′	
p6	*aadA-*XhoI 3′	5′-CCGCTCGAGTTATTTGCCGACCTACCTTGG-3′	
p7	*guaA*compF-SalI	5′-GCGTCGACGGTTTATAGCTAGATCTTTTGATTTGGC-3′	
p8	*guaA*compRC-SalI	5′-GCGTCGACTCAGCAGAATTTGCAGATGTATTCCC-3′	
p9	*guaA F*	5′-GATTTTGGATCCCAATATAGCC-3′	
p10	*guaA R*	5′-CCCATTCTATGGTTGATGGAGGCTTAGA-3′	
p11	*kan 5′-*NdeI	5′-CATATGAGCCATATTCAACGGGAAACG-3′	
p12	*kan term* XbaI	5′-GCTCTAGACTAGCGCCGTCCCGTCAA-3′	
p13	*guaA16159-*SalIF	5′-GTCGACGGATGGAATTGTAGGCCG-3′	
p14	*guaA15926-*SalIRC	5′-GTCGACGTAAACACTGGATTGTTGCGC-3′	
p15	*flgB*_*Po*_-XhoI	5′-TAATACTCGAGCTTCAAGGAAGATTT-3′	
p16	*aacC1*-NheI	5′-GCTAGCCGATCTCGGCTTGAACG-3′	

One possible explanation for these results is that the location of the antibiotic-resistance cassette in strain *guaA*::*kan* did not inactivate the N-terminal GATase domain of GuaA, and this truncated gene product conferred an enzymatic activity not present in the wild-type strain (Fig. 5A). The function of the GATase domain in *guaA* is to liberate ammonia from glutamine and transfer it to the enzyme's ATP-pyrophosphatase domain for conversion of XMP to GMP (Abbott *et al*., 2006). In the absence of the second domain, it is possible that residual activity of the truncated protein was somehow responsible for the anomalous data observed with this mutant. To test this, a *guaAB* deletion mutant was constructed by replacing the *guaAB* coding region with a spec/strep-resistance cassette through allelic exchange. The deletion of the *guaAB* operon was confirmed by PCR (Fig. 5A). This mutant, designated B31A3 68-1 Δ*guaAB,* was engineered to eliminate the entire *guaAB* operon, including the coding region for the GATase catalytic domain that was present in the original *guaA*::*kan* mutant. When B31A3 68-1 Δ*guaAB* cells were grown in the presence of [^3^H]-Hyp, no radioactivity was detected in the purified DNA or RNA (Fig. 4A). These results suggested that the GATase catalytic domain was most likely responsible for the spurious activity observed in the original *guaA*::*kan* mutant. Therefore, we elected to use the Δ*guaAB* mutant in these biochemical studies.

The Δ*guaAB* mutant was complemented by cloning a wild-type copy of *guaAB* into the shuttle vector pBSV2G and this construct, or the empty shuttle vector, was introduced into the Δ*guaAB* mutant (Table 3). These strains were then tested for incorporation of [^3^H]-Hyp as described above. When clone Δ*guaAB*/pBSV2G*guaAB* was grown in BSKII containing [^3^H]-Hyp, radioactivity was detected in both RNA and DNA (∼32 and 28 fmol ng^−1^ nucleic acid respectively) (Fig. 4A). As a control, the deletion mutant containing an empty shuttle vector was grown in the presence of [^3^H]-Hyp. In these cells, no radioactivity was detected (Fig. 4A). These data indicated that the incorporation of the [^3^H]-Hyp was indeed due to the presence of the *guaAB* operon. It should be noted that strain Δ*guaAB*/pBSV2G*guaAB* cells incorporated higher levels of [^3^H]-Hyp than did the wild-type bacteria. This result could be due to the increased copy number (5–10 copies per cell) of pBSV2G*guaAB* in these cells (Tilly *et al*., 2006). The purified DNA isolated from strains B31A3 68-1 and Δ*guaAB*/pBSV2G*guaAB* grown in BSKII containing [^3^H]-Hyp was degraded and analysed by HPLC to determine the specific DNA base containing the [^3^H]-label. In both cases, the incorporated [^3^H]-Hyp was converted into dGuo (data not shown). These results indicated that the *guaAB* operon was required for incorporation of Hyp into the nucleic acids of *B. burgdorferi* and that dGuo is the sole DNA product of Hyp metabolism in this bacterium. These data also demonstrate that *B. burgdorferi* was unable to convert Hyp to AMP, confirming the lack of a functional PurAB (adenylosuccinate synthase/lyase) for the conversion of IMP to AMP as suggested by previous genomic and Southern blot analyses (Fraser *et al*., 1997; Barbour *et al*., 2005; Pettersson *et al*., 2007).

### IMP dehydrogenase possesses secondary substrate activity for dIMP

**Fig. 6:**
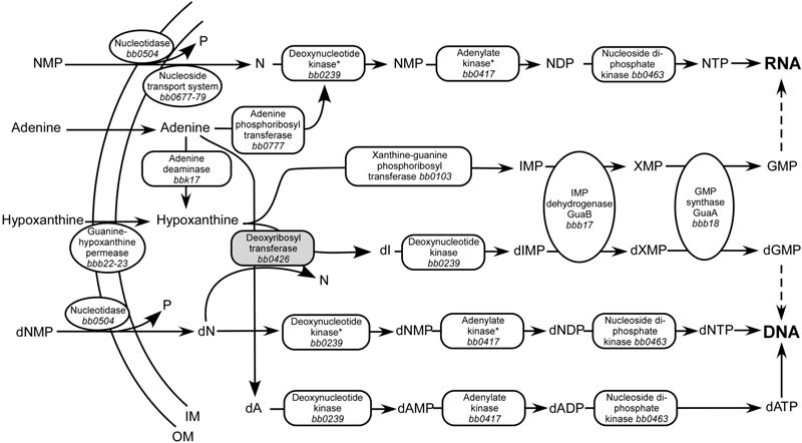
Proposed purine salvage pathway for *B. burgdorferi.* This diagram represents a proposed pathway for purine salvage based upon the experimental data presented in this report and genome data annotated in ERGO (Fraser *et al*., 1997; Overbeek *et al*., 2003). The deoxyribosyltransferase, BB0426, is shaded to highlight its pivotal role in this pathway. Genes assigned putative activities for non-specific substrates are designated with an asterisk. These assignments were based on activities reported for homologous genes in the BRENDA database (Chang *et al*., 2009).

The *guaB* gene in *B. burgdorferi* has been previously shown to encode a functional inosine-5′-monophosphate dehydrogenase (Zhou *et al*., 1997). The reported activity of the inosine-5′-monophosphate dehydrogenase in *B. burgdorferi* is the catalysis of IMP oxidation to XMP with a corresponding reduction of NAD^+^ to NADH. In organisms containing an active RNR, the GuaAB pathway serves the dual purpose of producing guanine nucleotides for RNA (GTP) and after enzymatic reduction of GDP by RNR and subsequent phosphorylation, dGTP for DNA synthesis. In the absence of an active RNR, the mechanism for dGTP production in *B. burgdorferi* was not immediately obvious. Our preliminary [^3^H]-Hyp incorporation data suggested a critical role for GuaAB in the purine salvage pathway and DNA synthesis in *B. burgdorferi*. Furthermore, the incorporation data strongly suggested that in addition to the previously published activity, *B. burgdorferi* GuaB could also catalyse the conversion of dIMP to dXMP. To determine if GuaB could catalyse this conversion, the activity of GuaB from *B. burgdorferi* was examined when purified recombinant protein was combined with dI, dIMP or IMP substrates. When dIMP or IMP was used as a substrate in the reaction mixture, the conversion of dIMP to dXMP or IMP to XMP was detected and the *K*_m_ calculated to be 66 μM or 208 μM respectively. Additionally, no activity was detected when dI was used as a substrate, indicating that the phosphorylation of inosine or dI to IMP or dIMP, respectively (presumably by *dgk*), was required before conversion to XMP or dXMP. The ability of GuaB to convert dIMP to dXMP provided a critical link for the multistep conversion of Hyp to dGTP and subsequent utilization for DNA synthesis. Based upon the data presented, we propose a novel pathway for purine salvage that circumvents the need for a RNR in *B. burgdorferi* that includes the transport of deoxynucleotide precursors, the activity of a deoxyribosyltransferase and the concerted activities of GuaAB (Fig. 6).

## Discussion

The genome of *B. burgdorferi* contains few genes encoding enzymes for *de novo* biosynthesis of amino acids, fatty acids or nucleobases and thus, it must rely on its arthropod vector or various vertebrate hosts to provide these ‘essential’ components of cellular macromolecules. While the utilization of peptides/amino acids for protein synthesis and fatty acids for glycolipids, lipids and lipoproteins synthesis have been partially defined (reviewed in Gherardini *et al*., 2009), little is known about the requirements for nucleobases in *B. burgdorferi.* Typically, bacteria lacking the *de novo* synthesis of bases must rely on ‘salvage’ pathways for the utilization of bases and/or nucleosides for RNA and DNA synthesis. In general, these pathways include enzymes for the conversion of bases to nucleosides for RNA synthesis (or energy storage, e.g. ATP or GTP) or to reduce NDPs or NTPs to deoxynucleotides (catalysed by RNRs) for DNA synthesis.

Cross-genome comparisons between the Lyme disease spirochaete and the relapsing fever spirochaetes (*B. hermsii, B. recurrentis* and *B. duttonii*), which encode an operon for a class Ib RNR (Barbour *et al*., 2005; Pettersson *et al*., 2007; Lescot *et al*., 2008), did not identify similar gene(s) in *B. burgdorferi* (Barbour *et al*., 2005; Pettersson *et al*., 2007). While we and others were unable to detect an RNR homologue in *B. burgdorferi*, it was still possible that *B. burgdorferi* might harbour proteins with similar enzymatic activity but divergent in gene sequence. This possibility was tested experimentally using the incorporation of the radio-labelled pyrimidine ribonucleotide CMP. When cells were grown in the presence of [^3^H]-CMP, radioactivity was only detected in the RNA, not the DNA, indicating that *B. burgdorferi* was not able to enzymatically catalyse the reduction of the ribonucleotides (Fig. 1, column labelled CMP). Thus without a RNR, it seemed likely that *B. burgdorferi* required host-derived sources of deoxynucleosides, as well as nucleosides, for growth.

Like *B. burgdorferi*, *U. urealyticum* does not encode an RNR operon, suggesting that this bacterium is dependent upon the uptake of deoxypurines and deoxypyrimidines from the host (Glass *et al*., 2000). Subsequent studies have demonstrated that *U. urealyticum* does, in fact, transport purine and pyrimidine deoxynucleosides and after phosphorylation by thymidine or deoxyadenosine kinase, incorporate these precursors into DNA (Carnrot *et al*., 2003). *Bacillus subtilis* and *Bacillus mojavensis* have also been shown to require deoxynucleosides for DNA synthesis when the organisms are grown anaerobically (Folmsbee *et al*., 2004). Because the class Ib RNR in *Bacillus* requires oxygen to be catalytically active, the bacterium must rely upon uptake of deoxynucleosides for DNA replication when the cells are grown in the absence of oxygen (Folmsbee *et al*., 2004). Thus, it is clear that some bacterial species can survive and replicate by scavenging exogenous deoxynucleosides in lieu of a functional RNR.

When purine substrates were tested in the nucleotide incorporation assay, it appeared that *B. burgdorferi* was capable of generating reduced purine ribonucleotides (Fig. 1, column labelled AMP and GMP). In addition, purine deoxynucleosides (dAdo and dGuo) were incorporated into RNA (Fig. 1, column labelled dAMP and dGMP), suggesting nucleotide phosphorylase activity. These observations suggested biochemical deviations from the classic salvage pathway and led to a series of experiments to understand these experimental data. Bioinformatics revealed an open reading frame in *B. burgdorferi* (*bb0426*) that putatively encoded a protein with active site residues similar to a 2′-deoxyribosyltransferase (Fig. 2) (Anand *et al*., 2004; Finn *et al*., 2006). Furthermore, microarray analysis has demonstrated that *bb0426* is constitutively expressed in *B. burgdorferi* (J.A. Boylan, unpubl. data), suggesting that the gene is transcribed during mid-log phase of growth. N-deoxyribosyltransferases (DRTase) are represented by two classes of enzymes: DRTase I enzymes catalyse the transfer of 2′-deoxyribose from a purine deoxynucleoside to a purine base exclusively, while DRTase II enzymes are able to transfer a 2′-deoxyribose from any deoxynucleoside donor to pyrimidines, purines or between pyrimidine and purine bases (Kaminski, 2002). The structure and functions of these enzymes have been well characterized in *Lactobacillus* species. *Lactobacillus helveticus* harbours genes encoding both a DRTase I (also designated as Ptd) and a DRTase II (also designated as Ntd) (Kaminski, 2002; Anand *et al*., 2004). As *Lactobacillus* species contain functional RNRs, it seems likely that the primary function of the DRTases in this bacterium is to recycle 2′-deoxynucleosides to maintain nucleotide pools for DNA synthesis. It is interesting to note that *bb0426* homologues were identified in the genomes of Lyme disease spirochaetes while no homologues were found in the genomes of relapsing fever *Borrelia*, *Treponema* or *Leptospira,* all of which harbour genes encoding RNRs.

Kinetic studies with purified recombinant BB0426_opt_ demonstrated similar function and activity to those reported for *L. helveticus* DRTase I (Table 1) (Kaminski, 2002)*.* Purified *B. burgdorferi* BB0426_opt_ was able to transfer a deoxyribosyl moiety from dAdo to Gua or from dGuo to Ade yielding dGuo or dAdo respectively. However, BB0426_opt_ was not able to transfer the deoxyribose group from deoxypyrimidines or deoxypurines to pyrimidine nucleobases, suggesting that it had activity similar to *L. helveticus* DRTase I, not DRTase II. It is interesting to note that deoxypurine monophosphates did not act as deoxyribose donors, suggesting that once the deoxynucleosides were phosphorylated, they were committed to incorporation into DNA. The enzymatic activity of BB0426 reported here provided an explanation for the radiolabel incorporation of AMP, GMP, dAMP and dGMP observed in *B. burgdorferi* (Fig. 1). For example, dAMP would be dephosphorylated, transported into the cell and BB0426 would transfer the deoxyribosyl moiety from dAdo to Gua yielding dGuo and Ade. Ade would then be converted to Ado by adenine phosphoribosyltransferase (Apt, encoded by *bb0777*) and to ATP by Ndk (encoded by *bb0463*) for incorporation into RNA (Fig. 6). Likewise, AMP could be dephosphorylated to Ado, transported into the cell and phosphorylated to ATP for RNA synthesis (Fig. 6). Additionally, Ado could be hydrolysed to Ade and ribose 5-phosphate by purine nucleoside phosphorylase (putatively encoded by *bb0588, bb0375* and/or *bbI06*) and Ade could then be converted to dAdo by BB0426 and to dATP for DNA synthesis. Based upon experimental data presented here, it is likely that *B. burgdorferi* utilizes BB0426 (with the activities of enzymes outlined in Fig. 6) for purine salvage in lieu of a RNR to maintain sufficient levels of purine ribonucleosides and deoxynucleosides for RNA and DNA synthesis.

Additionally, and perhaps as important, purified BB0426_opt_ was shown to transfer the deoxyribosyl moiety from either dAdo or dGuo to Hyp yielding dI. Previously, Pettersson *et al*. (2007) reported that *B. burgdorferi* could incorporate Hyp into RNA and DNA. The activities of BB0426 reported here could explain these data. Following conversion of Hyp to dI by BB0426, it is likely that, after phosphorylation to dIMP, GuaB converts dIMP to dXMP, which could then be converted to dGMP by GMP synthase (GuaA). Indeed, we were able to experimentally establish that purified, recombinant GuaB converted dIMP to dXMP. Moreover, analyses of DNA isolated from cells labelled with [^3^H]-Hyp showed that >99% of the label was incorporated into dGuo (Fig. 4B). These results demonstrated that Hyp can be utilized via two pathways for incorporation into nucleic acids (Fig. 6). First, following a typical purine salvage pathway, Hyp is converted to IMP by a phosphoribosyltransferase (Apt or Gpt), and to GMP by GuaAB for incorporation into RNA. Or second, Hyp can be ribosylated by BB0426, and after phosphorylation by deoxynucleoside kinase (Dgk), can be converted to dGMP by GuaAB for incorporation into DNA. This pathway illustrates how purine salvage in *B. burgdorferi* has evolved in the absence of a RNR. It is important to stress that for survival, *B. burgdorferi* must have an external source of deoxypurines and deoxypyrimidines for DNA synthesis. Additionally, if exogenous sources of dGMP are limited, or to supplement dGuo transport, the activities of BB0426, GuaA and GuaB provide an alternate pathway to generate dGTP from salvaged Hyp. This simple adaptation makes it possible for *B. burgdorferi* to colonize and successfully infect host tissues when guanine nucleotide precursors are at levels too low to support growth.

## Experimental procedures

### Bacterial strains and culture conditions

All *B. burgdorferi* strains used in this study are listed in Table 3 and are derived from strain B31 clone A3, which lacks the plasmid cp9 but harbours all 20 other plasmids described in the parental strain MI-B31 (Elias *et al*., 2002). Strain A3 68-1 was derived by introducing strain A3 into a mouse, followed by reisolation, plating and screening single colonies for loss of lp25 and lp56. *B. burgdorferi* and *B. hermsii* were grown in liquid BSKII medium supplemented with 6% rabbit serum (Barbour, 1984). *B. burgdorferi* was plated in solid BSK medium as previously described (Rosa and Hogan, 1992). All cultures were grown at 34°C with 2.5% CO_2_ and antibiotics were added as required (kanamycin: 200 μg ml^−1^, gentamicin: 40 μg ml^−1^ and streptomycin: 50 μg ml^−1^). *E. coli* strains were grown in Luria broth containing the appropriate antibiotics (kanamycin: 50 μg ml^−1^, ampicillin: 100 μg ml^−1^).

### Construction of the *guaAB* mutants

To construct the *guaA*::*flgB*_*p*_*-kan* mutant, the *guaA* gene was amplified from B31 genomic DNA using primers 9 and 10 (Table 3) and cloned into the vector pCR-XL-TOPO (Invitrogen, Carlsbad, CA), yielding p*guaA*. A 233 bp region of *guaA (*amino acids 185–264) was removed from p*guaA* by inverse PCR using the Expand Long PCR system (Roche, Indianapolis, IN) and primers 13 and 14 (Table 3), yielding linear p*guaA*_mut_ with SalI sites at its ends. The kanamycin-resistance cassette, *flgB*_p_-*kan* (Bono *et al*., 2000), was amplified from pBSV2 (Stewart *et al*., 2001) with XhoI ends, using primers 15 and 16 (Table 3), and cloned into the pCR2.1-TOPO vector (Invitrogen). The *flgB*_P_-*kan* gene cassette was removed from the pCR2.1-TOPO vector by XhoI digestion and ligated into inactivation constructs digested with SalI to create p*guaA*::*flgB*_P_-*kan*. Twenty micrograms of p*guaA*::*flgB*_P_-*kan* plasmid DNA purified from *E. coli* was transformed into A3-M9 as previously described (Samuels, 1995; Elias *et al*., 2002; Grimm *et al*., 2004) and the recombinants were selected in solid BSK medium containing kanamycin. Colonies were screened by PCR for the presence of the kanamycin-resistance cassette within the *guaA* locus, using primers 1 and 2 (Table 3). Total genomic DNA was prepared from PCR-positive A3-M9 *guaA*::*flgB*_P_-*kan* clones and screened with a panel of primers for the presence of all *B. burgdorferi* plasmids (Elias *et al*., 2002). A clone that retained the plasmid content of the parent strain was used in further experiments.

To construct the *B. burgdorferi*Δ*guaAB* mutant, the *guaAB* genes were deleted from the *B. burgdorferi* strain A3 68-1 by allelic exchange using the spectinomycin/streptomycin-resistance cassette, *flaB*_p_*-aadA* (Jewett *et al*., 2007a). Briefly, a 3.9 kbp fragment that included *guaAB* and 500 bp of flanking upstream and downstream sequence, was amplified from B31 strain A3 genomic DNA using the Expand Long PCR system (Roche) and primers 1 and 2 (Table 3). The PCR product was ligated into pCR-XL TOPO (Invitrogen) generating p*guaAB*. A 2.9 kbp *guaAB* DNA fragment was removed from p*guaAB* by inverse PCR using the Expand Long PCR system (Roche) and primers 3 and 4 (Table 3), yielding linear pΔ*guaAB* with SalI restriction sites at each end. The spectinomycin/streptomycin-resistance cassette, *flaB*_p_-*aadA* (Jewett *et al*., 2007a), was amplified using primers 5 and 6 (Table 3). The PCR product containing *flaB*_p_-*aadA* was treated with XhoI and ligated into SalI cut-pΔ*guaAB*, yielding pΔ*guaAB*::*aadA*. Twenty micrograms of pΔ*guaAB*::*aadA* plasmid DNA purified from *E. coli* was transformed into strain A3 68-1 as previously described (Samuels, 1995; Elias *et al*., 2002; Grimm *et al*., 2004) and the recombinants were isolated on solid BSK medium containing streptomycin. Colonies were screened by PCR to insure that the streptomycin-resistance cassette had replaced *guaAB* using either primer pairs 1 and 2 or 5 and 6 (Table 3). Total genomic DNA was prepared from PCR-positive A3 68-1 Δ*guaAB*:: *flaB*_p_-*aadA* strains and screened with a panel of primers for the presence of all *B. burgdorferi* plasmids (Elias *et al*., 2002). A strain that retained the *B. burgdorferi* plasmid content of the parent strain was used in further experiments.

### Complementation of the *ΔguaAB* mutant

The Δ*guaAB* mutant was complemented with pBSV2G *guaAB*, a shuttle vector carrying a wild-type copy of the *guaAB* operon and native promoter. Plasmid pBSV2G *guaAB* was constructed by PCR-amplifying a 3.4 kbp DNA fragment containing the *guaAB* operon and its putative promoter region with SalI ends from wild-type *B. burgdorferi* genomic DNA, using Vent polymerase (Invitrogen) and primers 7 and 8 (Table 3). TA ends were added to this DNA fragment using *Taq* polymerase and the product cloned into the vector TOPO-XL (Invitrogen). The 3.4 kbp *guaAB* DNA fragment was removed from TOPO-XL by SalI digestion and cloned into the *B. burgdorferi* shuttle vector pBSV2G (Elias *et al*., 2002) digested with SalI. The plasmid structure and sequence were analysed and verified by restriction digestion and sequence analysis. The *guaAB* mutant was transformed with the pBSV2G *guaAB* plasmid and positive transformants were selected as previously described (Jewett *et al*., 2007b). A strain that retained the *B. burgdorferi* plasmid content of the parent strain was used in further experiment.

### Cloning, overexpression and purification of BB0426

To characterize the putative 2′-deoxyribosyltransferase (BB0426), the native nucleotide sequence from *B. burgdorferi* was codon-optimized for expression in *E. coli* (Boylan *et al*., 2006) (sequence available upon request) and the synthetic gene, BB0426_opt_ (GeneArt, Burlingame, CA) was ligated into expression vector pET17b (Novagen, Madison, WI), which introduces a T7 epitope into the recombinant protein. The construct was transformed into *E. coli* strain BL21 pLysS (Novagen) and gene expression induced with IPTG. Cells expressing recombinant BB0426 _opt_ were lysed using a French pressure cell (12 000 psi) and cell debris was removed by centrifugation (10 000 × *g* for 15 min at 4°C). The supernatant was applied to a T7 Affinity column (Novagen) and purified protein was eluted as described in the manufacturer's protocol. The purity of BB0426_opt_ was estimated by SDS-PAGE, and protein concentration was determined using a BCA protein assay kit (Sigma-Aldrich, St Louis, MO).

### 2′-deoxyribosyltransferase *assay*

Purified recombinant BB0426_opt_ (1.5 μg) was added to a 1 ml reaction mixture containing 20 mM of a deoxyribose donor and 20 mM deoxyribose acceptor in 50 mM potassium phosphate buffer at pH 6.0. Reaction mixtures were incubated at 37°C and 230 μl aliquots were removed at 0, 5, 10 and 20 min intervals. Negative controls were prepared in the same manner, except that the enzyme was heat-inactivated at 95°C for 10 min. A standard curve was generated for each substrate and potential products tested at the following concentrations; 2.0, 5.0, 7.0 and 10.0 mM respectively. HPLC analyses were conducted on an Agilent 1200 system using a Supelcosil LC-18-S 4.6 mm × 150 mm, 5 μm analytical column (Supelco, Park Bellefonte, PA). Samples, standards and controls were filtered through a 0.45 μm membrane and 50 μl was injected onto the column equilibrated with 50 mM potassium phosphate, pH 4.5 containing 8% methanol. The products were eluted using an isocratic gradient for 20 min while UV absorbance was monitored at 254 nm. A unit of enzyme activity was defined as 1 nmol product generated per min.

### Nucleotide incorporation assay

Nucleotide incorporation assays were performed by growing *Borrelia* strains in 100 ml of BSKII containing 20 µCi (1 nmol) of tritium [^3^H]-labelled ribonucleoside-5′-monophosphates or deoxyribonucleoside-5′-monophosphates (Moravek, Brea, CA) to a density of ∼8 × 10^7^ cells ml^−1^. The cells were harvested by centrifugation (8000 *g,* 15 min at 4°C), washed 3× in HS buffer (50 mM NaCl in 10 mM HEPES, pH 7.4) and nucleic acids were isolated using Qiagen RNA/DNA Maxi Kit (Valencia, CA) per the manufacturer's instructions. The purity of RNA and DNA samples was verified by agarose gel electrophoresis and A_260/280_. Incorporation of labelled nucleotide was assayed by scintillation counts and normalized to the concentration of the nucleic acid. In addition, purity of each sample was confirmed by treating the RNA or DNA samples with DNase or RNase respectively, and repeating scintillation analysis.

### DNA digestion and HPLC analysis

DNA samples, prepared as described above, containing [^3^H]-labelled nucleotides were analysed to determine the fate of the radiolabel tag and nucleotide composition. A 50 μl aliquot of the DNA preparation was denatured (97°C for 2 min) and then immediately placed on ice. Ten microliters of 0.3M NaOAc pH 5.3, 5 μl 20 mM ZnSO_4_ and 10 μl P1 Nuclease (Sigma-Aldrich) were added to each sample and incubated at 37°C for 2 h. Next, 10 μl of 10× Antarctic phosphatase buffer and 2 μl (10 units) of Antarctic phosphatase (NEB, Ipswich, MA) were added and incubated at 37°C for 20 h. The reaction mixture was analysed by HPLC under the following conditions: 75 μl aliquot of sample was injected onto a Supelcosil LC-18-S 4.6 mm × 150 mm, 5 μm analytical column (Supelco) equilibrated with 50 mM potassium phosphate, pH 4.5, containing 8% methanol. Compounds were eluted from the column isocratically for 20 min at 1 ml min^−1^, monitoring absorbance at 254 nm. Furthermore, spectral data were collected for each peak using a diode array detector scanning from 200 to 600 nm. Peaks were identified by matching retention time with that of an authentic deoxynucleoside standard (dAdo, dC, dI, dGuo or dT), and further confirmation was made by matching corresponding spectral scans. If labelled DNA was used, fractions were collected corresponding to each eluted peak, utilizing an automated fraction collector, and the presence of radioactivity quantified in each peak fraction using a scintillation counter.

### IMP dehydrogenase assay (GuaB)

Recombinant IMP dehydrogenase (obtained from Dr Lizbeth Hedstrom, Brandeis University) was assayed as described by Zhou *et al*. (1997). Briefly, the reaction mixture contained 50 mM Tris buffer at pH 8.0, 100 mM KCl, 1 mM DTT, 1 mM NAD, 0.1 mM substrate and 0.002 mM purified enzyme (∼95% purity). All components (minus substrate) were incubated at 25°C at A_340_. Once UV absorbance stabilized, substrate (IMP, dIMP or dI) was added and the reaction mixture was monitored at A_340_ for 10 min.
